# Methanol in Plant Life

**DOI:** 10.3389/fpls.2018.01623

**Published:** 2018-11-09

**Authors:** Yuri L. Dorokhov, Ekaterina V. Sheshukova, Tatiana V. Komarova

**Affiliations:** ^1^N.I. Vavilov Institute of General Genetics, Russian Academy of Sciences, Moscow, Russia; ^2^A.N. Belozersky Institute of Physico-Chemical Biology, Lomonosov Moscow State University, Moscow, Russia

**Keywords:** cell wall, homogalacturonan, methanol, pectins, pectin methylesterase, pectin methylesterase inhibitor, plant immunity, stress

## Abstract

Until recently, plant-emitted methanol was considered a biochemical by-product, but studies in the last decade have revealed its role as a signal molecule in plant-plant and plant-animal communication. Moreover, methanol participates in metabolic biochemical processes during growth and development. The purpose of this review is to determine the impact of methanol on the growth and immunity of plants. Plants generate methanol in the reaction of the demethylation of macromolecules including DNA and proteins, but the main source of plant-derived methanol is cell wall pectins, which are demethylesterified by pectin methylesterases (PMEs). Methanol emissions increase in response to mechanical wounding or other stresses due to damage of the cell wall, which is the main source of methanol production. Gaseous methanol from the wounded plant induces defense reactions in intact leaves of the same and neighboring plants, activating so-called methanol-inducible genes (MIGs) that regulate plant resistance to biotic and abiotic factors. Since PMEs are the key enzymes in methanol production, their expression increases in response to wounding, but after elimination of the stress factor effects, the plant cell should return to the original state. The amount of functional PMEs in the cell is strictly regulated at both the gene and protein levels. There is negative feedback between one of the MIGs, aldose epimerase-like protein, and *PME* gene transcription; moreover, the enzymatic activity of PMEs is modulated and controlled by PME inhibitors (PMEIs), which are also induced in response to pathogenic attack.

## Introduction

Plants are the source of over a million metabolites (Cragg and Newman, 2013), including methanol, which was described back in 1661 by Robert Boyle as “sowrish spirit” in boxwood pyrolysis (Boyle, 1965). Originally, the out-dated name of methanol – wood alcohol – indicated its exceptional plant origin, but currently, it is known that endogenous metabolic methanol found in the human body can be not only a product of the plant diet but also a product of the life of microorganisms of the gastrointestinal tract and the processes of methylation-demethylation of DNA, RNA and proteins (Dorokhov et al., 2015). The source of endogenous plant methanol, as in the case of mammals, can be the process of demethylation of nucleic acids and proteins, but a cell wall pectin demethylesterification reaction is added to this process (Dorokhov et al., 2012).

**FIGURE 1:**
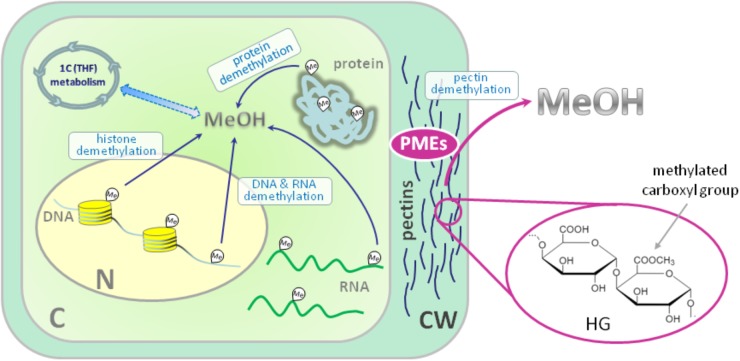
Sources of endogenous methanol in plant cell. The main impact to overall cellular methanol production is made by pectin methylesterases (PMEs) in the reaction of cell wall pectin demethylesterification. PMEs digest ester bonds in the methylated homogalacturonan (HG) component of pectin releasing methanol. The other source of methanol in plant cell is the demethylation of such macromolecules as DNA, RNA and proteins. Methanol carbon could be included in cellular metabolic pathways: formaldehyde and formic acid formed from methanol make a contribution via the folate-driven one-carbon (1C) cycle in the biosynthesis of serine, methionine, as well as purines and thymidylate in nucleic acids. C, cytoplasm; N, nucleus; CW, cell wall; HG, homogalacturonan; MeOH, methanol; Me, methyl group; PMEs, pectin methylesterases; THF, tetrahydrofolate. Histones are designated with yellow cylinders.

In general, the source of metabolic methanol formation in plants is the demethylation of macromolecules (Figure 1). If the biochemical processes of methylation and demethylation of RNA, DNA and protein leading to methanol formation in animals and plants have a common mechanism (Dong et al., 2014), the demethylation of cell wall pectins with the participation of pectin methylesterases (PMEs) is purely a plant or microbial process (Dorokhov et al., 2015). In general, the polysaccharides of the primary cell wall of plants consist of cellulose, hemicellulose and pectins, which form a gel-like matrix, determine cell adhesion, porosity and stiffness (Saffer, 2018). Polysaccharides of dicots and non-graminaceous monocots include polymers of galacturonic acid and are represented by three main types: homogalacturonan (HG) (65% of total pectins), rhamnogalacturonan-I (RG-I) (20–35%) and rhamnogalacturonan-II (RG-II) (10% of pectins) (Atmodjo et al., 2013). HG is a homopolymer of (1–4)-α-d-galacturonic acid (D-GalA) in which up to 80% of carboxyl groups can be methylated (Figure 1).

Pectin methylesterases of higher plants encoded by a multigenic family (67 putative isoforms in Arabidopsis) (Wang et al., 2013) and are synthesized as precursor proteins that are cleaved at the stage of precursor delivery through the endoplasmic reticulum to the cell wall (Markovic et al., 2002; Louvet et al., 2006) with the removal of the leader sequence (Dorokhov et al., 2006). Although it is believed that the contribution of the processes of plant DNA and histone methylesterification/demethylesterification in the total release of methanol into the atmosphere by plants is negligible (Fall and Benson, 1996), these processes are important for understanding the epigenetic modifications of the plant genome (Gan et al., 2015; Zhang et al., 2018).

Methanol′s role could be very significant, especially in transcriptional control, for example, via the regulation of the DNA methylation statuses of *Arabidopsis thaliana RESISTANCE METHYLATED GENE 1* and *WRKY22* in response to biotic stress (Deleris et al., 2016). In contrast to the participation of methanol in epigenetic processes, studies on the participation of pectins and PMEs in the formation of methanol in recent years have been devoted to the important role of methanol in plant development and in its response to stress effects (Dorokhov et al., 2012, 2015; Komarova et al., 2014a,b).

Here, we will consider the participation of methanol in plant life and its involvement in growth processes and the manifestation of protective responses against pathogens and adverse environmental factors.

## Methanol Participation in Plant Growth and Development

The participation of methanol in growth and development is determined by the function of pectins in the formation of the cell wall. Pectin polysaccharides are synthesized in the *cis*-, medial- and *trans*-Golgi (Sandhu et al., 2009; Atmodjo et al., 2013; Kim and Brandizzi, 2014, 2016). Numerous enzymes of pectin biosynthesis located in the Golgi apparatus were isolated and described, and their transmembrane type II topology assuming absence of a cleavable signal sequence was proved (Atmodjo et al., 2013). Methyl esterification of pectins probably occurs simultaneously with its synthesis (Atmodjo et al., 2013). With the help of secreted vesicles moving mainly along the actin cytoskeleton, pectins are delivered in a highly methylesterified form to the cell wall, which is building up at the stage of formation of the cell plate separating two new cells (Daher and Braybrook, 2015). The role of pectins in plant growth and development is evidenced by the phenotypes of Arabidopsis mutants and transgenic lines, in which the synthesis and modification of individual pectin polysaccharides is disrupted (Atmodjo et al., 2013; Saffer, 2018).

Plants with altered HG length also have multiple developmental defects, including an increased number of petals, variable phyllotaxis and early flowering, indicating that HG mechanical properties affect different developmental processes (Xiao et al., 2014, 2017). Mutants that affect pectin synthesis suffer from dwarfism and have decreased cell lengthening (Krupková et al., 2007; Saffer et al., 2017).

**FIGURE 2:**
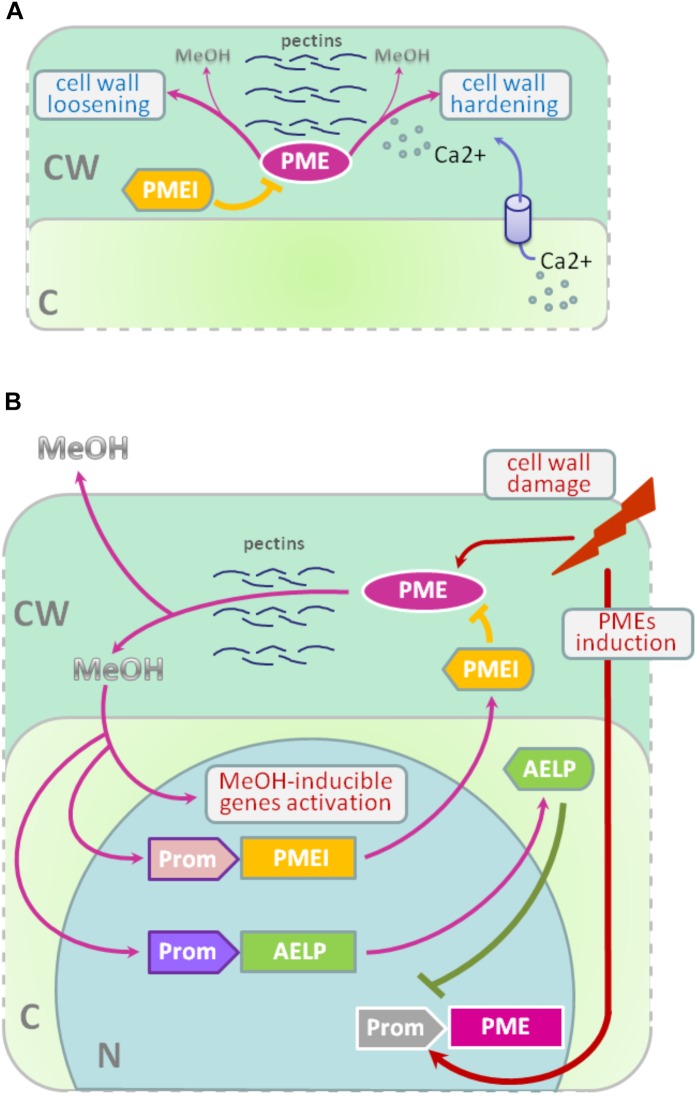
PME-PMEI and PME-AELP feedback during growth and after stress impact. **(A)** Modification of cell wall as a result of the coordinated action of PMEs and PMEIs. Demethylesterification is accompanied by cross-linking of HG molecules with Ca^2+^ ions, resulting in the strengthening of the cell wall, for example, during pollen tube growth. However, pectin demethylesterification can trigger the process leading to cell-wall loosening in apical meristem and hypocotyl, which is highly important for the shift from isotropic to anisotropic growth. **(B)** In fully expanded source leaves, PME activity is low but significantly increases under stress conditions, especially during the mechanical damage of tissues or pathogen attacks. As a result, de-esterification processes are accelerated and methanol emissions are dramatically increased. Methanol, in turn, activates methanol-inducible genes (MIGs), including aldose-epimerase-like protein (AELP), which is involved in intercellular transport and possibly controlling the transport and metabolism of sugars. Moreover, AELP negatively regulates *PME* gene transcription, making the cell return to a normal state after the end of the stress impact. Methanol-mediated coordination of defense reactions is based on the feedback mechanism: when excess methanol is released, via its action on AELP and PMEIs, it lowers the synthesis and activity of PME and returns the methanol emission rate to pre-stress state. C, cytoplasm; CW, cell wall; N, nucleus; PME, pectin methylesterase; PMEI, PME inhibitor; AELP, aldose epimerase-like protein; Prom, promoter region for *AELP* (purple), *PMEI* (pink) or *PME* (gray) genes; MeOH, methanol.

Methyl esterification of HG is a key determinant of cell wall properties, growth and leaf position (Peaucelle et al., 2008, 2012; Müller et al., 2013; Daher and Braybrook, 2015; Levesque-Tremblay et al., 2015; Hocq et al., 2017). Since most HG is synthesized in the methyl esterified form, during the growth of the cells it can be demethylesterified by the action of the PME enzymes, which are in turn regulated by a pectin methylesterase inhibitors (PMEIs) (Wolf et al., 2009; Jolie et al., 2010). After demethylesterification, HG can form Ca^2+^-pectate-cross-linked complexes, referred to as “eggboxes,” which strengthen the cell wall (Peaucelle et al., 2012; Daher and Braybrook, 2015). This process is observed, for example, during *Nicotiana tabacum* pollen tubes growth (Holdaway-Clarke et al., 2003; Bosch et al., 2005). On the other hand, increased PME activity correlates with the accumulation of demethylesterified HG in the Arabidopsis hypocotyl and apical meristem and leads to the cell wall loosening essential for growth symmetry breaking (Peaucelle et al., 2008, 2011, 2015; Figure 2A).

Pectin methylesterases interact with PMEIs to influence fruit development and ripening (Reca et al., 2012). Moreover, there are correlations between PME activity, the level of pectin de-esterification and L-ascorbic acid production in the later stages of tomato fruit ripening (Rigano et al., 2018).

The participation of PMEs in the demethylesterification of HG inevitably leads to the formation of methanol. As a consequence, methanol emissions from plant leaves are much higher when the leaves are young and expanding than when they reach maturity (Nemecek-Marshall et al., 1995; Galbally and Kirstine, 2002; Oikawa et al., 2011). This phenomenon can also be observed in developing tobacco leaves at the stage of their transition from the state of acceptors (sink-leaves) of photoassimilates to the state of donors (source-leaves) (Burch-Smith and Zambryski, 2012). A similar sink-source modification occurs with the participation of tobacco PME producing methanol from pectins (Komarova et al., 2014a). When studying the role of methanol in the functioning of plasmodesmata in tobacco sink-leaves, an increased content of *PME* (EMBL Accession #AJ401158) transcripts (Dorokhov et al., 2006) and raised concentration of methanol in the sap and tissues of immature leaves was found. Moreover, the genes for sieve element occlusion protein (SEOP), auxin-repressed protein (ARP), salicylic acid binding catalase (SABC) involved in tobacco plant growth and signal transmission proved to be methanol-sensitive (Komarova et al., 2014a).

Methanol, formed during the demethylesterification of HG, appears to also have a beneficial effect on the overall cellular metabolism. It is known that spraying plant leaves with 10–50% methanol can stimulate the photosynthetic activity and overall productivity of C3-plants (Nonomura and Benson, 1992). Although the mechanism responsible for this phenomenon is uncertain, it is assumed that formaldehyde and formic acid formed from methanol make a contribution via the folate-driven one-carbon (1C) cycle in the biosynthesis of serine, methionine, as well as purines and thymidylate in nucleic acids (Hanson and Roje, 2001). Moreover, [^13^C]-labeled methanol was shown to enter cells of higher plants (*Acer pseudoplatanus*) in suspension culture and to be metabolized to [3-^13^C]serine, [^13^CH_3_]methionine, and [^13^CH_3_]phosphatidylcholine in addition to the induction of the methyl-β-d-glucopyranoside *de novo* synthesis (Gout et al., 2000).

## Methanol Emission as a Stress Signal and Its Feedback Control

Because the cell wall separates and protects the cell from the environment (Malinovsky et al., 2014), the PMEs contained therein play a significant role in this defense mechanism (Komarova et al., 2014b). In addition to the direct effects of PMEs on tobacco cell wall, these enzymes also act via methanol released through the demethylesterification of pectins. Mechanical damage (Dorokhov et al., 2012) and the attack of pathogens (von Dahl et al., 2006; Körner et al., 2009) increase the synthesis of tobacco PMEs, accelerate de-esterification processes and dramatically increase methanol emission (Lionetti et al., 2012; Komarova et al., 2014b; Dorokhov et al., 2015). Methanol is considered not only a by-product of PME activity but also a participant in cell signaling and an inducer of plant protection reactions (Tran et al., 2018). Transgenic tobacco plants with overexpression of the *PME* genes from *A. thaliana* or *Aspergillus niger* dramatically increased methanol emission and resistance to plant sap sucking pests *Myzus persicae* (aphid) and *Bemisia tabaci* (whitefly) (Dixit et al., 2013). Moreover, the released methanol can act as a signaling molecule that induces the defense responses of both the native plant and the leaves of the neighboring plant (Dorokhov et al., 2012). It has been suggested that methanol functions as a DAMP-like alarm signal, and it probably functions as an elicitor-active DAMP in monocot grasses (Hann et al., 2014). The signal function of methanol is to control the so-called MIGs that regulate plant resistance to abiotic and biotic factors (Dorokhov et al., 2012). Among the MIGs, are genes encoding aldose 1-epimerase-like protein (mutarotase) (AELP), PMEI and β-1,3-glucanase, involved in controlling the intercellular transport of macromolecules and creating favorable conditions for the reproduction of viruses but preventing bacterial colonization (Dorokhov et al., 2012, 2015; Komarova et al., 2014b). Moreover, methanol released into the air after a leaf injury results in a “priming” effect on intact leaves, setting the stage for the within-plant and neighboring plant immunity (Dorokhov et al., 2012). While the mechanism (direct or indirect) of the cellular impact of an increase of physiological methanol is unclear, it can be assumed that one of methanol′s functions is to control its own synthesis.

In general, prolonged and continuous exposure to stress factors, such as prolonged mechanical wind impact, accompanied by the synthesis of PMEs, causes irreversible changes in woody plants (Hamant and Moulia, 2016) like coniferous trees on Atlantic coastal cliffs (Jaffe and Forbes, 1993). Short-term effects do not lead to the significant changes and depletion of cellular biosynthetic resources (Bostock et al., 2014), since there is a mechanism for returning the cell to its original state when the PMEs synthesis and methanol emission are downregulated after the elimination of the stress factor impact. One of the key players of such control and regulation are PMEIs. For example, during *A. thaliana* infection by the necrotrophic pathogen *Botrytis cinerea*, PME activity and pectin demethylesterification are dynamically modulated by PMEIs (Lionetti et al., 2017).

Recently, another PME activity controlling mechanism was identified: the methanol-inducible gene *AELP* involved in intercellular transport and possibly controlling the transport and metabolism of sugars (Sheshukova et al., 2017) negatively regulates tobacco *PME* expression. The proposed model for the feedback regulation of gene expression involving *PMEI* and *AELP* suggests that when the cell wall is mechanically damaged, the *PME* genes are activated (Figure 2B); synthesis of PME and its secretion in the tobacco cell wall cause the active synthesis and emission of methanol, leading, in particular, to an increase in its concentration in the cytoplasm. This process is accompanied by induction of the *PMEI* genes and activation of the expression of the *AELP* gene through its methanol-inducible promoter. As shown in Figure 2B, AELP is capable of suppressing the transcription activity of the tobacco *PME* gene (Sheshukova et al., 2017) and reducing the level of methanol emission, which ultimately leads to a decrease in the transcription of the tobacco *AELP* gene. In accordance with this scheme, AELP proteins are likely to behave as transcription factors by inhibiting the expression of the *PMEs* in the nucleus and not in the apoplast.

Thus, the proposed model assumes the regulation of PMEs functioning at both the gene and protein levels.

## Conclusion

Recent research has strengthened our view that endogenous metabolic methanol in plants not only is a by-product of biochemical processes of plant life but also performs an important signaling function. Influencing genes involved in the plasmodesmata gating and the intercellular transport of macromolecules, methanol participates in the controlled growth and development of plants (Dorokhov et al., 2012). Since the process of the maturation of cells after their division is accompanied by the essential PMEs-mediated demethylesterification of the cell wall HG, this process inevitably leads to the formation of methanol, which affects the function of the genes involved in the development of the plant. Excess synthesis of methanol, for example, due to overexpression of *PME*, which has been observed in transgenic tobacco plants (Hasunuma et al., 2004; Sheshukova et al., 2017), leads to dwarfism. Thus, methanol seems to be necessary for the coordinated work of the participants in the plant development program. The same methanol-mediated coordination is observed during the development of plant protective reactions in response to abiotic and biotic stresses. Methanol as a gaseous substance is released into the air after a violation of the integrity of the cell wall and has a “priming” effect on intact leaves, as if preparing them for a possible attack by the aggressor (Dorokhov et al., 2012). At the heart of methanol-mediated coordination of defense reactions lies the feedback mechanism: when excess methanol is released, it acts on tobacco *AELP* (Sheshukova et al., 2017) and *PMEI* (Lionetti et al., 2017), lowering the synthesis and activity of PME and returning the methanol emission rate to a pre-stress state.

## Author Contributions

YD, ES, and TK conceived the concept, prepared, revised, and finalized the manuscript. All the authors read and approved the manuscript.

## Conflict of Interest Statement

The authors declare that the research was conducted in the absence of any commercial or financial relationships that could be construed as a potential conflict of interest.
